# Biofilm-derived bile duct microbiota in liver transplantation: high-quality genomes of *Klebsiella pneumoniae*, *Enterococcus faecalis*, and *Enterococcus faecium*

**DOI:** 10.1128/mra.00886-25

**Published:** 2026-01-22

**Authors:** Anna Baborski, Oliver Rohland, Tia Wuenschmann, Michael Bauer, Rosalind J. Allen, Anne Busch

**Affiliations:** 1 Theoretical Microbial Ecology, Friedrich Schiller University Jena 9378, Jena, Germany; 2 Department of General, Visceral and Vascular Surgery, Jena University Hospital 718002, Jena, Germany; 3 Cluster of Excellence Balance of the Microverse, Friedrich Schiller University Jena 9378, Jena, Germany; 4 Department of Anaesthesiology and Intensive Care Medicine, University Hospital Jena 232632, Jena, Germany; University of Pittsburgh School of Medicine, Pittsburgh, Pennsylvania, USA

**Keywords:** biofilm, liver transplant patients, *Klebsiella*, *Enterococcus*, catheter

## Abstract

We present draft genomes of *Klebsiella pneumoniae (K. pneumoniae*), *Enterococcus faecalis* (*E. faecalis*), and *Enterococcus faecium* (*E. faecium*) isolated from a bilioenteric catheter after liver transplantation. Genome sizes were 5.58 Mb, 2.95 Mb, and 2.78 Mb, with G+C contents of 57.25%, 37.6%, and 37.99%, respectively, highlighting biofilm-associated bile duct colonizers.

## ANNOUNCEMENT

Liver transplant patients receive antibiotic treatment and immunosuppressive medication to prevent organ rejection (1, 2). However, this can lead to an increased risk of infection. *Enterobacteriaceae* and *Enterococcaceae* are the most prevalent families within biliary diseases (3, 4). We describe the genetic characteristics of *Klebsiella pneumoniae* (MS-Stent-001-342), *Enterococcus faecalis* (MS-Stent-001-343), and *Enterococcus faecium* (MS-Stent-001-344), three bacterial isolates from bilioenteric catheters from a liver transplant recipient. These strains were successfully used to establish an *in vitro* model for studying infection and bacterial interactions. The catheter, collected post-surgery from a liver transplant patient as part of the MS-Stent study at Jena University Hospital, was removed, rinsed, and processed with ultrasonication to detach the biofilm. Subsequently, the biofilm was stored in PBS or in MAST CRYOBANK at –70°C. For isolation, MS-Stent-001-342 was cultured on MacConkey Agar (Carl Roh, Karlsruhe, Germany). Meanwhile, MS-Stent-001-343 and MS-Stent-001-344 were grown on Enterococcus Selective Agar (NutriSelect, Darmstadt, Germany). All strains were incubated for 24 h at 37°C under aerobic conditions.

Each strain was cultured in BHI medium for 24 h at 37°C. To perform gDNA isolation, the protocol of the PureLink Genomic DNA Kit (Thermo Fisher Scientific, 2024) was followed. Here, 20 µL of ≥45,000 U lysozyme (50 mg/mL) was added to each sample instead of the lysis buffer to increase cell lysis. Using the multiplex PCR approach, neither of the isolated *Enterococcaceae* strains contains VanA or VanB resistance genes (5). This approach targets *vanA*, *vanB*, *vanC1*, *vanC2/C3*, *E. faecalis*, *E. faecium,* and *rrs* (16S rRNA) with primers as described before (5).

Following the gDNA isolation, strain specification was done using 16S gene sequencing by EUROFINS (Ebersberg, Germany) as previously described (6), identifying MS-Stent-001-342 as *K. pneumoniae*, MS-Stent-001-343 as *E. faecalis* and MS-Stent-001-344 as *E. faecium* using NCBI BLASTn (7).

Whole genome short read sequencing of all three strains was performed on the iSeq100 platform with a 300-cycle iSeq reagent kit generating paired-end (2 × 150 bp) raw reads. Here, libraries were prepared using standard protocols for the Illumina DNA Prep Kit and the Nextera CD indexes. A long-read sequencing library was prepared with a one-dimensional ligation sequencing kit SQK-LSK109 (Oxford Nanopore Technologies [ONT]) and NEBNext Companion Module for ONT Ligation Sequencing kit (New England BioLabs, USA) following each manufacturer’s protocol. Subsequently, the library was loaded into a FLO-MIN106 flow cell (R9.4.1, ONT) on a MinION sequencer (ONT). Base-calling of the fast5 reads was done using MinKNOW (v.19.10.1 [ONT]) with Guppy v.3.3.3 in high-accuracy base-calling mode.

FastQC was used for read quality assessment (v.0.74) (8). *De novo* assembly of short reads was performed with SPAdes (v.3.15.5) (9), while long-read sequence *de novo* assembly was done using Flye (v.2.9.5) (10). Hybrid assemblies were then polished using pilon (v.1.20.1) (11). Subsequently, species identification on genome assemblies was done using NCBI BLASTn (7). The genome assemblies were checked using QUAST (v.5.3.0) (12), and annotations were performed with Prokka (v.1.14.6) (13). The multi-locus sequence types (MLSTs) of all three species were determined using MLST online service from Center for Genomic Epidemiology from the Technical University of Denmark (14). Contamination was checked with EZbio ContEST16S (15). Unless otherwise noted, default parameters were used for all software (Table 1).

**TABLE 1:** Genomic characteristics of bilioenteric catheter-derived isolates on hybrid genomes

Characteristic	Value or result for bacterial strain:		
	MS-Stent-001-342	MS-Stent-001-343	MS-Stent-001-344
Species-level identification from genome assemblies	*K. pneumoniae*	*E. faecalis*	*E. faecium*
MLST	1,630	21	Unknown
Illumina raw reads	2,229,530	2,163,742	2,402,168
ONT raw reads, average length of ONT reads (bp)	149,581, 6,004.0 ± 8,240.2	266,093, 5,184.6 ± 6,491.6	376,061, 3,877.6 ± 5,522.4
Average read coverage of hybrid assemblies	58.0 ± 12.0	105.2 ± 24.2	208.6 ± 114.5
DNA size (bp)	5,581,245	2,946,259	2,782,127
G+C content (%)	57.25	37.6	37.99
Contig count	3	5	17
Largest contig	5,321,311	2,762,364	795,579
Contig N50 (bp)	5,321,311	2,762,364	447,908
Contig L50 (bp)	1	1	3
Total protein-encoding genes predicted	5,118	2,757	2,700
rRNA	25	12	3
tRNA	86	61	58
tmRNA	1	1	1
Contamination	Not contaminated	Not contaminated	No contamination detected

## Data Availability

Sequencing data for this study have been deposited in the NCBI. The BioProject PRJNA1280976 contains three BioSamples: SAMN49540934 (accession JBPKJC000000000, SRA runs SRR34394232 and SRR34394233), corresponding to *Klebsiella pneumoniae* MS-Stent-001-342; SAMN49540972 (accession JBPKJD000000000, SRA runs SRR34395282 and SRR34395283), corresponding to *Enterococcus faecalis* MS-Stent-001-343; and SAMN49541016 (accession JBPKJE000000000, SRA runs SRR34395232 and SRR34395233), corresponding to *Enterococcus faecium* MS-Stent-001-344.

## References

[1] Shbaklo N, Tandoi F, Lupia T, Corcione S, Romagnoli R, De Rosa FG. 2022. Bacterial and viral infections in liver transplantation: new insights from clinical and surgical perspectives. Biomedicines 10:1561. doi:10.3390/biomedicines10071561 35884867 PMC9313066

[2] Hernandez MDP, Martin P, Simkins J. 2015. Infectious complications after liver transplantation. Gastroenterol Hepatol (N Y) 11:741–753. 27134589 PMC4849501

[3] Dondorf F, Graf M, Deeb AA, Rohland O, Felgendreff P, Ardelt M, Settmacher U, Rauchfuss F. 2023. Pathogen detection in patients with perihilar cholangiocarcinoma: implications for targeted perioperative antibiotic therapy. Hepatobiliary Pancreat Dis Int 22:512–518. doi:10.1016/j.hbpd.2022.01.005 35153139

[4] Eberhardt N, Santamarina BG, Enghardt M-L, Rohland O, Hussain I, Tannert A, Thieme L, Rubio I, Arndt H-D, Bauer M, Busch A, Jürgen Rödel, Bettina Löffler. 2024. The effects of photoactivated ciprofloxacin and bile acids on biofilms on bile duct catheters. Int J Antimicrob Agents 63:107086. doi:10.1016/j.ijantimicag.2024.107086 38218325

[5] Kariyama R, Mitsuhata R, Chow JW, Clewell DB, Kumon H. 2000. Simple and reliable multiplex PCR assay for surveillance isolates of vancomycin-resistant enterococci. J Clin Microbiol 38:3092–3095. doi:10.1128/JCM.38.8.3092-3095.2000 10921985 PMC87194

[6] Dumann G, Rohland O, Abdel-Glil MY, Allen RJ, Bauer M, Busch A. 2024. Draft genomes of the bile duct microbiome strains Klebsiella pneumoniae and Enterococcus lactis isolated from bilioenteric drainages with biofilm-forming abilities. Microbiol Resour Announc 13:e0020224. doi:10.1128/mra.00202-24 39470240 PMC11636323

[7] Altschul SF, Gish W, Miller W, Myers EW, Lipman DJ. 1990. Basic local alignment search tool. J Mol Biol 215:403–410. doi:10.1016/S0022-2836(05)80360-2 2231712

[8] Babraham Bioinformatics. 2011. FastQC: a quality control tool for high throughput sequence data

[9] Bankevich A, Nurk S, Antipov D, Gurevich AA, Dvorkin M, Kulikov AS, Lesin VM, Nikolenko SI, Pham S, Prjibelski AD, Pyshkin AV, Sirotkin AV, Vyahhi N, Tesler G, Alekseyev MA, Pevzner PA. 2012. SPAdes: a new genome assembly algorithm and its applications to single-cell sequencing. J Comput Biol 19:455–477. doi:10.1089/cmb.2012.0021 22506599 PMC3342519

[10] Kolmogorov M, Yuan J, Lin Y, Pevzner PA. 2019. Assembly of long, error-prone reads using repeat graphs. Nat Biotechnol 37:540–546. doi:10.1038/s41587-019-0072-8 30936562

[11] Walker BJ, Abeel T, Shea T, Priest M, Abouelliel A, Sakthikumar S, Cuomo CA, Zeng Q, Wortman J, Young SK, Earl AM. 2014. Pilon: an integrated tool for comprehensive microbial variant detection and genome assembly improvement. PLoS One 9:e112963. doi:10.1371/journal.pone.0112963 25409509 PMC4237348

[12] Gurevich A, Saveliev V, Vyahhi N, Tesler G. 2013. QUAST: quality assessment tool for genome assemblies. Bioinformatics 29:1072–1075. doi:10.1093/bioinformatics/btt086 23422339 PMC3624806

[13] Seemann T. 2014. Prokka: rapid prokaryotic genome annotation. Bioinformatics 30:2068–2069. doi:10.1093/bioinformatics/btu153 24642063

[14] Larsen MV, Cosentino S, Rasmussen S, Friis C, Hasman H, Marvig RL, Jelsbak L, Sicheritz-Pontén T, Ussery DW, Aarestrup FM, Lund O. 2012. Multilocus sequence typing of total-genome-sequenced bacteria. J Clin Microbiol 50:1355–1361. doi:10.1128/JCM.06094-11 22238442 PMC3318499

[15] Lee I, Chalita M, Ha S-M, Na S-I, Yoon S-H, Chun J. 2017. ContEst16S: an algorithm that identifies contaminated prokaryotic genomes using 16S RNA gene sequences. Int J Syst Evol Microbiol 67:2053–2057. doi:10.1099/ijsem.0.001872 28639931

